# The proteomic profile of a mouse model of proliferative vitreoretinopathy

**DOI:** 10.1002/2211-5463.12252

**Published:** 2017-06-29

**Authors:** Bernadett Márkus, Zsuzsanna Pató, Zsolt Sarang, Réka Albert, József Tőzsér, Goran Petrovski, Éva Csősz

**Affiliations:** ^1^ Department of Biochemistry and Molecular Biology Faculty of Medicine University of Debrecen Hungary; ^2^ Department of Ophthalmology Faculty of Medicine University of Szeged Hungary; ^3^ Department of Ophthalmology Oslo University Hospital and University of Oslo Norway

**Keywords:** 2D electrophoresis, animal model, mass spectrometry, PVR, TG2

## Abstract

Proliferative vitreoretinopathy (PVR) develops as a complication of retinal detachment surgery and represents a devastating condition leading to serious vision loss. A good animal model that permits extensive functional studies and drug testing is crucial in finding better therapeutic modalities for PVR. A previously established mouse model, using dispase injection, was analyzed from the proteomic point of view, examining global protein profile changes by 2D electrophoresis, image analysis and HPLC–tandem mass spectrometry‐based protein identification. The easy applicability of the mouse model was used to study the role of transglutaminase 2 (TG2) in PVR formation by proteomic examination of dispase‐induced TG2 knockout vitreous samples. Our data demonstrate that, despite the altered appearance of crystallin proteins, the lack of TG2 did not prevent the development of PVR.

**Abbreviations**
2‐DE: 2D gel electrophoresis
Ctrl: control
ECM: extracellular matrix
FA: formic acid
IPG: immobilized pH gradient
KO: knock‐out
LC: liquid chromatography
MMP: matrix metalloprotease
MS/MS: tandem mass spectrometry
MS: mass spectrometry
OCT: optical coherence tomography images
PVR: proliferative vitreoretinopathy
RPE: retinal pigment epithelium
TG2: transglutaminase 2
WT: wild‐type

Proliferative vitreoretinopathy (PVR) develops as a complication in 8–25% of patients undergoing primary retinal detachment surgery. It is a multifactorial disease induced by a variety of factors 1. A hallmark of PVR is the aggressive proliferation of glial and retinal pigment epithelium (RPE) cells originating from retinal breaks or holes, making PVR an unsolved challenge. The epithelial‐to‐mesenchymal transition activated by different serum‐derived or vitreal factors is responsible for the formation of epiretinal membrane and hence PVR pathogenesis 2, 3. Recently, we have shown activation of neural progenitor cells in human eyes with PVR near the pars plana region, from which only the glial population seemed to respond to retinal injury by targeted migration into the vitreous 4.

Extracellular matrix (ECM) deposition in PVR appears with the concomitant release of matrix‐degrading enzymes including matrix metalloproteases (MMPs) with a likely function to prevent excess protein deposition 5.

Transglutaminase 2 (TG2), the most widely studied member of the transglutaminase family 6, has various functions: it can participate in the apoptosis process; it can be present on the cell surface and in the ECM; and it can modulate tissue stability and cell–matrix interactions 7, 8. TG2 plays an important role in wound healing; its absence may cause delayed wound closure that, to a certain extent, can be valuable in reducing scar formation 9, 10. Transglutaminases are expressed in many types of normal ocular tissues 11, 12 and TG2 is present in human PVR membranes. In PVR membranes, TG2 crosslinking of ECM proteins could prevent their degradation by the elevated protease activities and therefore may have an important role in the onset of PVR 5.

Although a number of proteins were identified in normal human vitreous 13, 14 and the potential contribution of genetic components to PVR has been recently described by some research groups, only the alterations of a few proteins and genes has been clarified in PVR formation 15, 16. 2D gel electrophoresis (2‐DE) coupled with mass spectrometry (MS)‐based protein identification, the widely used proteomic tool for the analysis of protein profile changes 17, has been applied to study the human vitreal proteins characteristic of PVR formation, and the presence of extracellular proteins, such as zinc finger protein 670 and prostaglandin D2 synthase, was demonstrated 18. In human vitreous samples originating from patients with PVR, 516 proteins have been identified by MS and increased levels of extracellular proteins and reduced levels of cytoskeletal proteins have been observed 19.

Considering the serious sight‐threatening feature and high incidence of PVR, there is increasing pressure to develop better medications for the treatment of this eye condition. In order to facilitate drug discovery, various animal models have been generated in the past. Most of the models induce PVR in rabbit, rat or piglet eyes either by injecting cells or by performing very invasive surgical procedures such as retinotomy 20, 21, 22. Our group used a suitable mouse model for PVR by injecting dispase into mouse eyes, since it is fast, reproducible and avoids use of more invasive surgical approaches 4. The presence of PVR in mice was demonstrated by imaging techniques in our wider collaborative network 4, but the exact proteomic signature was not examined in detail.

In this work, as a continuation of the previous study, we have analyzed the vitreal protein profile of dispase‐treated mice in order to examine the protein profile changes. A good animal model is increasingly important to elucidate the exact molecular pathomechanism of the PVR and can help foster drug discovery and drug testing providing suitable drug candidates for successful PVR therapy.

## Materials and methods

All chemicals used for 2‐DE and MS were of electrophoresis or liquid chromatography (LC)–MS grade and were purchased from Sigma‐Aldrich (St. Louis, MO, USA) unless stated otherwise.

### Animal samples

A mouse model of PVR was used by applying an intravitreal injection of a proteolytic enzyme, dispase. This model is known to induce glial activation as well as both epi‐ and subretinal membrane formation 23, 24. All animal experiments were performed according to the Association for Research in Vision and Ophthalmology (ARVO) Statement for the Use of Animals in Ophthalmic and Vision Research. Study protocols were approved by the Animal Care Committee of the University of Debrecen. Female 4‐ to 6‐month‐old wild‐type (WT; C57/BL6, *n* = 6) and TG2KO mice (*n* = 6) were anesthetized with pentobarbital (90 mg·kg^−1^, i.p.), and also received one drop of 1% procaine hydrochloride (Novocaine, EGIS, Budapest, Hungary) for local anesthesia and one drop of tropicamide (Mydrum, Chauvin Aubeans, Montpellier, France) for iris dilation. Four microliters of dispase (Sigma‐Aldrich; 0.4 U·μL^−1^, dissolved in sterile physiological saline solution) was injected intravitreally into the right eyes under stereomicroscopic control (Ctrl) using an automatic pipette fitted with a 30G 1/6 needle, as previously described 23. Ctrl animals received 4 μL of sterile physiological saline solution. Stratus optical coherence tomography images (OCT; Carl Zeiss Meditec, Dublin, CA, USA) were taken following injections to confirm PVR induction and monitor disease progression (results not shown here). Ctrl and dispase‐treated mice were sacrificed at the 14th day following injections when signs of PVR formation were evident: presence of epiretinal membrane and/or retinal detachment on OCT examination.

### Sample preparation and purification

The vitreous bodies of mice were isolated after guillotine removal of the cornea together with a scleral galler using a scalpel blade, followed by removal of the lens. After solubilization of the vitreous body with lysis buffer (pH 8.5) containing 7 m urea, 30 mm Tris, 2 m thiourea and 4% CHAPS, the lysates were sonicated in an ice‐cold water bath for 5 min and centrifuged at 16 900 ***g*** for 10 min at 4 °C. The supernatants were transferred to LoBind Eppendorf tubes and purified by Ready‐Prep 2‐D CleanUp Kit (Bio‐Rad Laboratories, Hercules, CA, USA) according to the manufacturer's protocol. Briefly, following precipitation and centrifugation, the pellets were dried and resuspended in 240 μL rehydration buffer containing 7 m urea, 2 m thiourea, 4% w/v CHAPS, 1% dithiothreitol (DTT), 2% v/v Bio‐Lyte (Bio‐Rad) and 0.001% bromophenol blue, and used immediately for isoelectric focusing.

### 2D gel electrophoresis

Three vitreous samples originating from each group (WT Ctrl, WT PVR, TG2 knock‐out (KO) Ctrl, TG2 KO PVR) were subjected to 2‐DE. First immobilized pH gradient (IPG) strips with an IPG (24 cm, pH 4–7; Bio‐Rad) were rehydrated with extracted vitreous proteins using passive rehydration at 20 °C overnight. This was followed by isoelectric focusing performed by applying 300 V for 3 h, which was gradually increased to 3500 V in 5 h and then held at 3500 V for 18 h. After isoelectric focusing, the IPG strips were immediately placed at −70 °C until equilibration. The IPG strips were equilibrated for 15 min in equilibration buffer (500 mm Tris/HCl, pH 8.5, 6 m urea, 2% SDS, 20% glycerol) containing 0.6% DTT and for 15 min in equilibration buffer containing 1.2% iodoacetamide. In the second dimension, the strips were laid on top of 12% polyacrylamide gels and covered with agarose. Using a Protean Plus Dodeca Cell (Bio‐Rad) the electrophoresis was carried out at 100 mA per gel for 24 h until the bromophenol blue dye reached the bottom of the gel. All 12 gels were run together under the same conditions. Proteins were stained using in‐house prepared ruthenium II tris(bathophenanthroline disulfonate) fluorescent dye 25 and gel images were recorded using Pharos FX Plus Molecular Imager (Bio‐Rad). Three biological replicates were analyzed in all cases, the samples from the Ctrl and the dispase‐treated groups were processed together on the same day.

### Quantitative analysis of protein expression profile using Delta2D software

Protein spot patterns were evaluated with delta2d (Decodon, Greifswald, Germany) software version 4.4. Briefly, the gel images were grouped as follows: (a) dispase‐treated WT (WT PVR), (b) physiological saline‐treated WT (WT Ctrl), (c) dispase‐treated TG2 KO (TG2 KO PVR) and (d) physiological saline‐treated TG2 KO (TG2 KO Ctrl). Gel images of three biological replicates were included in each group having altogether 12 images. Two data analysis sets were created: (a) in the first set, the WT Ctrl vs. WT PVR gel images were studied, while (b) in the second set, the TG2 KO Ctrl vs. TG2 KO PVR gel images were examined. Protein spot patterns of gels from each of the Ctrl and PVR groups were matched using the exact mode matching protocol and the group warping strategy. Using union mode, a fused image containing all spots present on all of the gels included in the data analysis set was generated. Every spot on each gel was quantified and the total quantity of the spots was considered as 100. The spot intensity was normalized according to the total intensity of all spots in each gel and was given as normalized spot volume (%V) compared with the total intensity. The mean of the normalized volume was calculated in the PVR group and divided by the mean of the normalized volume over the Ctrl group and the fold change values were calculated.

### Statistical analysis

The quantification table was generated by the Delta2D software and the level of significance was determined automatically using Student's *t* test. Those spots were considered significant where the *P* < 0.05 criterion was valid.

### In‐gel digestion

The protein spots showing significantly different intensities upon dispase treatment were excised manually from the 2D gels using a pipette tip and were digested in‐gel with trypsin. First, the gel spots were cut into 1 × 1 mm pieces and were destained using a 1 : 1 ratio of 25 mm ammonium bicarbonate pH 8.5 and 50% acetonitrile, followed by reduction with 20 mm DTT for 1 h at 56 °C. The alkylation was performed using 55 mm iodoacetamide for 45 min at room temperature in the dark, followed by digestion with 100 ng stabilized MS grade trypsin (ABSciex, California, USA) overnight at 37 °C. The reaction was stopped by adding formic acid (FA). The tryptic peptides were extracted from the gel pieces, dried in a vacuum concentrator and kept at −20 °C until MS analysis.

### Protein identification by HPLC–tandem mass spectrometry

For protein identification, the samples were redissolved in 10 μL 1% FA, separated on Easy nLCII (Bruker, Coventry, UK) nanoHPLC and analyzed using a 4000QTRAP (ABSciex) mass spectrometer. The chromatographic separation was performed at a flow rate of 300 nL·min^−1^ in a 90 min water/acetonitrile gradient. The mobile phase A was 0.1% FA in LC‐MS grade water (Sigma), while the mobile phase B was acetonitrile with 0.1% FA. The peptide mixture was loaded onto a 5 mm × 0.3 mm Zorbax 300SB desalting column packed with 5 μm particle size C18 resin (Agilent Technologies, Santa Clara, CA, USA). Thereafter, the peptides were separated on a 150 mm × 75 μm Zorbax 300SB analytical column (300 Å particle size, 3.5 μm pore size C18 resin; Agilent).

The tandem mass spectrometry (MS/MS) analysis was performed in positive ion mode and the information‐dependent acquisition method was applied. For protein identification, collision‐induced dissociation spectra were obtained in enhanced product‐ion mode at a scan rate of 4000 amu·s^−1^ and the rolling collision energy was utilized with the maximum of 80 eV. After the first mass scan (mass range 400–1700 amu), the charge state of the precursor ions was established by enhanced resolution and the MS/MS spectra of the two most intensive ions were recorded (mass range 100–1900 amu). The cycle time was 5.5 s, the spray voltage was 2800 V, the ion source gas was 50 psi, the curtain gas was 20 psi and the source temperature was 70 °C.

For protein identification based on the recorded MS/MS spectra, the proteinpilot 4.5 software (ABSciex) and the UniProtKB/Swiss‐Prot database (version 2014.06.11, 545536 entry) was used. The minimum criteria for protein identification were the presence of two peptides per protein with at least 95% confidence.

## Results

### Proteins characteristic for PVR induction upon dispase treatment in WT mice

**Figure 1:**
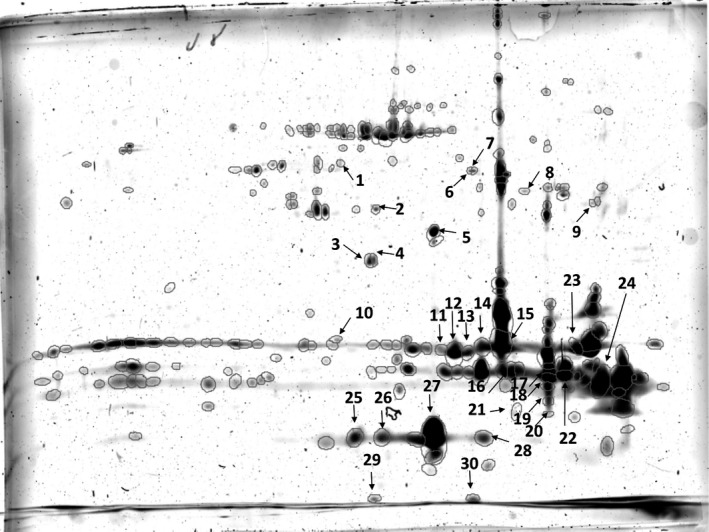
Fused image generated by the superposition of WT Ctrl and WT PVR gel images using delta2d software. The numbered spots were found to have significantly different intensities between the groups.

To study the proteins involved in the pathogenesis of PVR in mice, WT C57/BL6 mice were used and PVR was induced by dispase injection. 2‐DE was carried out on vitreous samples originating from three Ctrl and three dispase‐treated WT mice. Gels were stained with homemade ruthenium II tris(bathophenanthroline disulfonate) 25 and the gel images were analyzed using delta2d (Decodon) software. The WT Ctrl group was created from the three gel images originating from physiological saline‐treated samples and the WT PVR group was created from the three gel images originating from dispase‐treated samples. A fused image was generated by superimposing all gel images, and altogether 698 spots could be detected on the fused image (Fig. 1). The intensity of each spot on each gel was determined and the fold change between the groups was calculated. The intensity of 30 out of 698 spots showed significant (*P* < 0.05) changes as a result of dispase treatment and these 30 spots were excised from the gel, subjected to in‐gel trypsin digestion and subsequent HPLC‐MS/MS‐based protein identification (Tables 1 and S1). The comparison between the WT Ctrl vs. WT PVR group aimed to get information about the proteins for which altered appearance is associated with PVR.

**Table 1:** List of identified proteins in spots showing significantly changed intesity upon dispase injection in WT mice. The name of the identified protein, the spot number and fold change (WT PVR vs. WT Ctrl) value are indicated

Spot ID	Protein ID	Protein name	Fold change	*P*
WT1	P99024	Tubulin β‐5 chain	−0.23	0.03
WT2	Q04447	Ckb	−0.27	0.03
WT3	P62874	Guanine nucleotide‐binding protein G(I)/G(S)/G(T) subunit β‐1	−0.11	0.02
WT4	P62874	Guanine nucleotide‐binding protein G(I)/G(S)/G(T) subunit β‐1	−0.08	0.03
WT5	P24622	α‐Crystallin A chain	−0.23	0.02
WT6	O35737	Hnrnph1	−0.09	0.049
WT7	O35737	Hnrnph1	−0.11	0.04
WT8	P17182	Eno1	−0.21	0.049
WT9	P15105	Glul	−0.41	0.049
WT10	P14602	Hspb1	−0.32	0.02
WT11	P02525	Cryba1	−0.17	0.01
	P62696	Crybb2		
WT12	P02525	Cryba1	−0.37	0.046
	P62696	Crybb2		
WT13	P62696	Crybb2	−0.46	0.01
	P02525	Cryba1		
	Q9JJV1	β‐Crystallin A2		
WT14	P02525	Cryba1	−0.37	0.04
	P62696	Crybb2		
WT15	P02525	Cryba1	−0.41	0.01
WT16	P24622	α‐Crystallin A chain	−0.51	0.02
	Q9JJV1	β‐Crystallin A2		
WT17	P24622	α‐Crystallin A chain	−0.50	0.001
	P02525	Cryba1		
WT18	P02525	Cryba1	−0.32	0.04
	P24622	α‐Crystallin A chain		
WT19	P02525	Cryba1	−0.25	0.01
WT20	P02525	Cryba1	−0.11	0.03
WT21	P23927	Cryab	0.27	0.02
WT22	O35486	Crygs	−0.34	0.01
	P04344	γ‐Crystallin B		
WT23	P62696	Crybb2	−0.36	0.0002
WT24	P2392	Cryab	−0.44	0.03
	O35486	Crygs		
	Q61597	γ‐Crystallin C		
WT25	P24622	α‐Crystallin A chain	−0.47	0.03
WT26	P24622	α‐Crystallin A chain	−0.28	0.02
WT27	P24622	α‐Crystallin A chain	−0.49	0.02
WT28	P24622	α‐Crystallin A chain	−0.25	0.02
WT29	Q9D1U0	Grifin	−0.53	0.003
WT30	Q05816	Fatty acid‐binding protein, epidermal	−0.25	0.004

In case of 19 spots, different forms of crystallins were identified. Crystallins are known as stress proteins, from which three main types, α, β and γ, can be distinguished. The number of spots containing α‐crystallin A was the highest (eight out of 19) and the amount of various forms of crystallins except α‐crystallin B (Cryab) decreased. The level of some intracellular proteins, such as α‐enolase (Eno1), tubulin, creatine kinase B‐type (Ckb), glutamine synthetase (Glul), G protein, heterogeneous nuclear ribonucleoprotein H (Hnrnph1), heat shock protein β‐1 (Hspb1), grifin (Grifin) and fatty‐acid binding protein 5, was reduced in the dispase‐treated WT samples compared with the physiological saline treated ones (Table 1). In case of spots 11, 12, 13, 14, 16, 17, 18, 22 and 24, more than one protein with similar peptide counts reflecting almost equal quantities of each protein was detected. Therefore, the combined effect of these proteins could be monitored in such cases, and no information on the contribution of each individual protein to the spot intensity changes could be observed.

### Proteins characteristic for PVR induction upon dispase treatment in TG2KO mice

**Figure 2:**
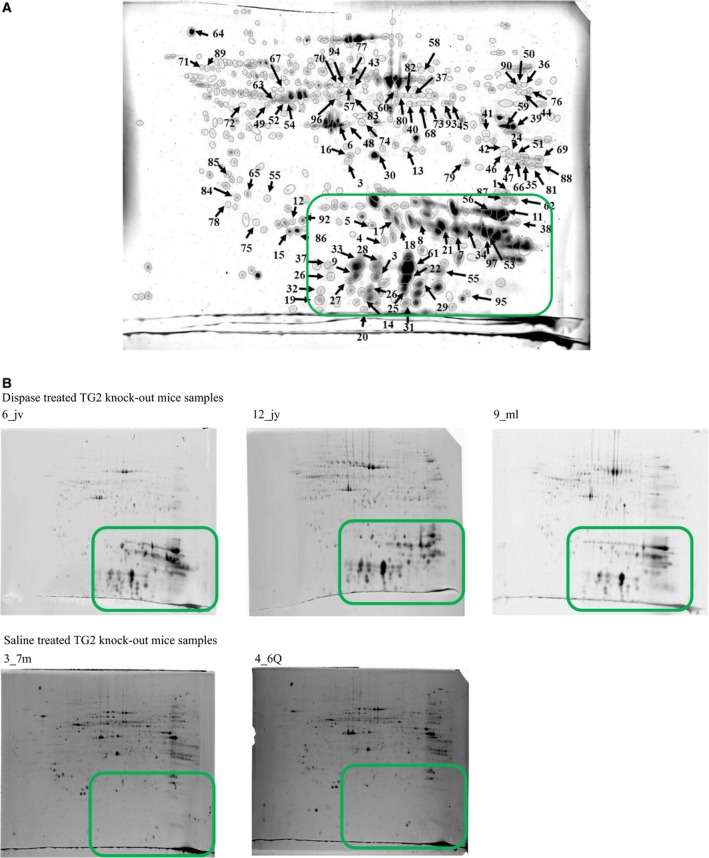
Fused image generated by the superposition of TG2KO Ctrl and TG2KO PVR gel images using delta2d software. (A) Image containing all spots. The numbered spots were found to have significantly different intensities between the groups. (B) Different spot pattern observed on the individual examined gels with emphasis on basic pH and lower molecular mass region.

Mice models are widely used to examine the function of well‐defined proteins as far as the KO animals for the studied protein can easily be created. Our group previously studied TG2, a multifunctional enzyme having diverse roles in different physiological and pathological conditions 26. TG2 has been shown to be present in PVR membranes 5, but the role of this enzyme in PVR has not been examined in detail. In order to demonstrate the feasibility of the mouse PVR model in elucidating the role of TG2 in PVR, dispase and physiological saline, respectively, were injected into the eyes of TG2 KO animals. The strategy for the study of WT Ctrl and WT PVR samples was applied in the case of TG2 KO samples as well; the vitreous samples were subjected to 2D electrophoresis followed by protein staining and image analysis. Due to a technical problem, on one of the gel images from the physiological saline‐treated TG2 KO mouse eyes very few spots could be detected, so this gel image was excluded from further analyses. The images from the TG2 KO Ctrl group created from the two gel images originating from physiological saline‐treated TG2 KO mouse eyes and from the TG2 KO PVR group created from the three gel images originating from dispase‐treated TG2 KO mouse eyes were superimposed to generate the fused image (Fig. 2). Altogether 866 spots were detected, out of which 97 showed significant (*P* < 0.05) changes in their intensities upon dispase treatment. The amount of proteins in 33 spots increased, while in 64 spots it decreased. Interestingly, some spots within the basic pH and lower molecular mass region of the gels could be detected only in case of TG2 KO PVR samples; in spite of otherwise good quality of protein separation, very few spots were visible in the gel region corresponding to basic, low molecular mass proteins in the case of the saline‐treated samples (Fig. 2B). The spots showing significantly different intensities were cut out and 37 of them were subjected to protein identification using HPLC‐MS/MS (Table S1).

**Figure 3:**
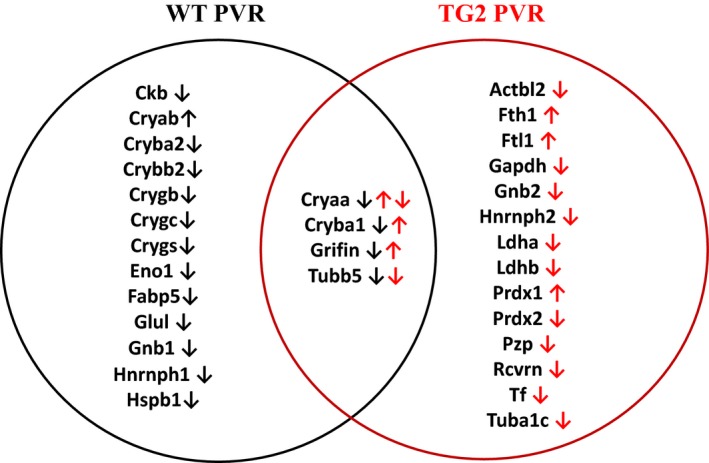
Summary of proteomic results of the proteins showing significant alterations in their amount upon dispase treatment. Black and red arrows show the direction of changes in WT and in TG2 KO samples, respectively, upon dispase treatment, compared with the physiological saline treated samples. The black arrows refer to the changes in the WT mice while the red arrows show the changes in the TG2 KO mice upon dipsase treatment. The abbreviations of protein names are used according to gene name. Cryaa, α‐crystallin A chain; Cryba2, β‐crystallin A2; Crygb, γ‐crystallin B; Crygc, γ‐crystallin C; Fabp5, fatty acid‐binding protein, epidermal; Ftl1, ferritin light chain 1; Gnb1, guanine nucleotide‐binding protein G(I)/G(S)/G(T) subunit β‐1; Gnb2, guanine nucleotide‐binding protein G(I)/G(S)/G(T) subunit β‐2; Hnrnph2, heterogeneous nuclear ribonucleoprotein H2; Ldha, l‐lactate dehydrogenase A chain; Ldhb, l‐lactate dehydrogenase B chain; Prdx1, peroxiredoxin‐1; Tuba1c, tubulin α‐1C chain; Tubb5, tubulin β‐5 chain.

In accordance with the previous results, most of the spots showing altered intensity changes upon dispase treatment contained α‐crystallin, but in this case, their level was increased upon the treatment (Table 2 and Fig. 3). The amount of tubulin, heterogeneous nuclear ribonucleoprotein and G‐protein was reduced upon dispase treatment, being in accordance with previous results obtained by the analysis of WT mice. The amount of the glycolytic enzyme glyceraldehyde‐3‐phosphate dehydrogenase, lactate dehydrogenase (Gapdh), cytoskletal protein β‐actin‐like protein 2 (Actbl2), recoverin (Rcvrn), peroxiredoxin 2, serotransferrin (Tf) and α‐2‐macroglobulin (Pzp) was also reduced. At the same time the level of Grifin, ferritin L and H chains and β‐crystallin A1 (Cryba1) increased in TG2 KO PVR samples. When the changes upon dispase injection in WT and TG2KO mice were compared (Fig. 3), among the studied proteins four of them were changed in both WT and TG2 KO mice. The level of tubulin β‐5 decreased upon dispase treatment independently of the presence of TG2, while the decrease in the level of Grifin, Cryba1 and α‐crystallin A observed in WT mice changed to an increase in the TG2 KO mice as a response to dispase injection. In the case of α‐crystallin A there was one spot where reduction in the protein amount could be detected in dispase‐treated TG2 KO mice.

**Table 2:** List of identified proteins in spots showing significantly changed intesity upon dispase injection in TG2KO mice. The name of the identified protein, the spot number and the fold change (TG2KO PVR vs. TG2KO Ctrl) value are indicated

Spot ID	Protein ID	Protein name	Fold change	*P*
TG2KO1	P99024	Tubulin β‐5 chain	−5.57	0.003
	P68373	Tubulin α‐1C chain		
TG2KO3	P24622	α‐crystallin A chain	76.45	0.01
TG2KO7	P70333	Heterogeneous nuclear ribonucleoprotein H2	−9.51	0.003
TG2KO14	P09528	Fth1	5.82	0.04
TG2KO15	P24622	α‐Crystallin A chain	−6.28	0.005
TG2KO16	Q8BFZ3	Actbl2	−7.01	0.01
TG2KO18	P02525	Cryba1	41.04	0.05
TG2KO19	P02525	Cryba1	5.13	0.01
TG2KO20	P24622	α‐Crystallin A chain	49.71	0.01
TG2KO26	P24622	α‐Crystallin A chain	128.66	0.02
TG2KO27	P02525	Cryba1	4.25	0.03
TG2KO31	P34057	Rcvrn	−18.90	0.02
TG2KO33	P16125	l‐Lactate dehydrogenase B chain	−7.59	0.007
TG2KO34	P24622	α‐Crystallin A chain	106.31	0.005
TG2KO38	Q61171	Prdx2	−7.51	0.001
TG2KO42	Q61838	Pzp	−20.54	0.0002
TG2KO44	P24622	α‐Crystallin A chain	196.24	0.006
TG2KO47	P29391	Ferritin light chain 1	56.65	0.04
TG2KO48	P24622	α‐Crystallin A chain	9.03	0.01
TG2KO49	P24622	α‐Crystallin A chain	17.14	0.02
	Q9D1U0	Grifin		
TG2KO50	P24622	α‐Crystallin A chain	8.92	0.02
TG2KO51	P24622	α‐Crystallin A chain	83.15	0.003
TG2KO55	P24622	α‐Crystallin A chain	31.65	0.03
TG2KO60	P16858	Gapdh	−13.74	0.0002
TG2KO64	P24622	α‐Crystallin A chain	42.28	0.0005
TG2KO67	P24622	α‐Crystallin A chain	18.79	0.02
TG2KO70	P24622	α‐Crystallin A chain	22.95	0.0005
TG2KO74	P24622	α‐Crystallin A chain	88.39	0.03
TG2KO77	P24622	α‐Crystallin A chain	31.74	0.005
TG2KO83	P62880	Guanine nucleotide‐binding protein G(I)/G(S)/G(T) subunit β‐2	−16.39	0.0001
TG2KO84	P24622	α‐Crystallin A chain	12.72	0.01
TG2KO87	P24622	α‐Crystallin A chain	21.14	0.0006
TG2KO88	P24622	α‐Crystallin A chain	36.78	0.0007
TG2KO90	P24622	α‐Crystallin A chain	11.36	0.004
	P02525	Cryba1		
	P35700	Peroxiredoxin‐1		
TG2KO91	P06151	l‐Lactate dehydrogenase A chain	−22.83	0.0005
TG2KO94	Q921I1	Tf	−7.40	0.0005
TG2KO96	P24622	α‐Crystallin A chain	23.98	0.0002

## Discussion

Based on current knowledge, many cell types and various factors appear to have a role in PVR pathogenesis. RPE cells exposed to the vitreous upon retinal detachment or retinal injury, migrate and proliferate giving rise to fibroblast‐like cells that are responsible for production of ECM components and a repair membrane 27, 28, 29. Collagen, fibronectin and growth factors are then produced by activated cells (Müller glia, macrophages, modified RPE cells) or originate from the serum itself to further enhance the proliferation and migration of RPE cells and hence the epithelial‐to‐mesenchymal transition – a key phenomenon in PVR formation 4, 29, 30.

In a mouse model of PVR the intravitreal injection of the proteolytic enzyme was used to activate RPE cells 23. Upon dispase treatment, the most prominent changes were related to different forms of crystallins. They function as chaperones, have protective roles 31 and their increased levels were found to be associated with intensive cell proliferation. Expression of Cryab has been found to be higher in tongue carcinoma compared with normal Ctrl tissue 32, overexpression of Cryab was found to be protective in retinal epithelial cells against stress‐induced apoptosis 33, while at gene expression level, the amount of α‐, β‐ and γ‐crystallin increased in the retina after injury 34, 35, 36. Secretion of Cryab in exosomes has recently been demonstrated 37, and thus it seems logical to have this protein released into the vitreous after retinal detachment, a phenomenon that often precedes the PVR formation. We here demonstrate for the first time increased levels of Cryab in the vitreous upon dispase treatment. It is not clear whether the increased crystallin level inhibits apoptosis of RPE cells via caspase 3 and 6 inhibition 38 and thus shifts the balance toward RPE cell proliferation or if the proliferating RPE cells produce and release more crystallin into the vitreous, which further enhances and sustains the RPE cell proliferation; in both scenarios, however, the result is a decreased apoptosis and increased RPE cell proliferation 33. In our study, the crystallins, especially α‐crystallin A, show an extensive post‐translational modification upon dispase treatment; almost half of the spots containing some forms of crystallin contained α‐crystallin A. There is evidence that in oxidative stress conditions following ischemia–reperfusion injury the amount and post‐translational modifications of crystallins are changed 38. The pathophysiological conditions leading to post‐translational modifications can only be speculated upon, hypothesizing the effect of MMPs and dispase, respectively, but to best of our knowledge, no experiments aiming to elucidate the phenomena behind the crystallin profile changes have been carried out.

The proteomic analysis of rat and rabbit PVR models have revealed the down‐regulation of collagen‐Iα1, α‐1‐antiproteinase and peroxiredoxin‐2 (Prdx2) in rabbit vitreous, and up‐regulation of different forms of α‐crystallin A, Tf, β‐crystallin B2 (Crybb2), matrix metalloproteinase‐3 and other proteins in rabbit retina 39, 40. The amount of the Eno1, vimentin, albumin and prohibitin was increased, while the amount of tubulin β‐2C, fructose‐bisphosphate aldolase A and other proteins was reduced in the retina of animals after retinal detachment 22. In the case of these models the PVR induction was done either by mechanical injury or by retinal detachment surgery and the examined sample was rabbit retina or vitreous 22, 39, 40.

The differentially expressed proteins upon dispase injection in WT mice, except Hnrnph1, Glul, ferritin H chain, Cryba1 and Grifin, were also found in human vitreous proteome 13. The level of Crybb2 and S was reduced in mice in accordance with published human data; using MS the above‐mentioned crystallin forms were detected in donors but not in patients with PVR 19. In human vitreous proteome of patients with proliferative diabetic retinopathy, a decrease in the amount of α‐crystallin A, β‐crystallin S (Crygs), tubulin β and creatin kinase B was observed 41 suggesting the possible implication of these proteins in the proliferative conditions; however, it is not known if their reduced level is the cause or the consequence of the proliferative state.

One of the methods used to examine the function of a gene or protein is by removing the gene by knocking it out from the genome 42. This KO technology can be used to study the function of various genes 43 and in our study we have chosen to examine the function of TG2 in PVR.

TG2, having transglutaminase, kinase and deamidase activities, can modify more than 300 different substrates 44, and can interact with numerous proteins (http://genomics.dote.hu/wiki) having a role in different physiological processes such as ECM remodeling 5 and wound healing 45. Transglutaminase activity is known to be present in the eye 46; as a protein crosslinker, TG2 may participate in cell adhesion in various ocular diseases such as allergic conjunctivitis 47, abrasion, cataract 48 and glaucoma 49. TG2 was found in PVR membranes and it was shown to modulate the phenotype and migration of RPE cells and Ctrl the ECM remodeling in PVR 5, 50. Our working hypothesis was that the crosslinking activity of TG2 may prevent the appropriate degradation of ECM proteins by the elevated protease activities, and therefore may contribute to the PVR development.

In order to study the role of TG2, a TG2 KO animal was created and PVR formation was examined in the absence of TG2. Interestingly, in spite of the marked protein profile changes (see below), OCT examinations suggested the formation of PVR (data not shown). It is not excluded, however, that TG2 may affect the timing of PVR formation but we do not have such information as we examined the already formed PVR condition only.

In the TG2 KO animals, the dispase treatment resulted in altered crystallin network; the α‐crystallin A and Cryab levels were increased. Both α crystallin A and B have been previously shown to be capable of inhibiting apoptosis 38, and they might supplement each other's function.

In the TG2 KO PVR samples, compared with the WT PVR samples, a different crystallin profile change was seen, yet the pathological outcome appeared to be the same at the time point of the analysis. TG2 has different substrates that it can bind to 44, 51, and therefore, depending on which one of these substrates is available for binding to it under different pathological conditions, it is not unexpected to induce an altered crystallin profile. These data indicate that more than one type of change in the profile of crystallins might be associated with PVR in mice. Crystallin changes have been observed in general in the case of proliferative diseases 31 and most probably the crystallin profile changes are not specific to PVR. According to the data presented in the literature, it cannot be judged if the crystallin profile changes are the cause or the consequence of a proliferative disease.

The crystallins identified in the different spots, having various pI values and sizes, most probably are the result of extensive post‐translational modifications affecting both the size and the net charge of the protein 52. Such modifications might result in spots showing opposite intensity changes containing the same protein. In TG2 KO PVR mice, the dispase injection led to increased α‐crystallin A amounts observed in spots 2, 9, 10, 14, 17, 19, 21, 22, 23, 25, 26, 27, 28, 29, 31, 32, 33 and 37 and decreased amounts observed in spot 5 indicating the presence of the extensive post‐translational modifications.

Taking into account that dispase is a protease and TG2 is a crosslinking enzyme generating protease‐resistant isopeptide bonds, one may speculate that in the absence of TG2, the proper crosslinks cannot be formed, which might result in increased crystallin cleavage by dispase. This may be the reason why some spots containing α‐crystallin are present in the lower molecular mass region of the TG2 KO PVR gels. The present study did not test this possibility; further research is needed to clarify the situation. It is likely that these differences will shine light on the different substrates that may play a role in the pathogenesis of PVR *in vivo*.

In the TG2 KO mice the dispase injection led to increased amounts of ferritin heavy and light chains. Ferritin maintains the iron homeostasis by limiting the level of Fe (II) able to participate in the formation of reactive oxygen species, thus protecting against oxidant toxicity 53. In the TG2 KO PVR samples, the intensity of ferritin light chain‐containing spots exceeded by almost 10 times the intensity of ferritin heavy chain (Fth1)‐containing spots, which can be indicative of oxidative stress.

## Conclusion

The examination of protein profile changes in the dispase‐induced mouse model of PVR by 2D electrophoresis followed by image analysis and subsequent HPLC‐MS/MS‐based protein identification has shown extensive changes related to crystallin proteins in dispase‐injected mice vitreous. To demonstrate the utility of this mouse model for functional studies, the dispase treatment was carried out on TG2KO mice and the model was used successfully to get more information on the protein profile changes in PVR in the lack of TG2. TG2 appears to be responsible for the remodeling of protein networks with alterations in stress management, indicating a possible protective role of TG2 in the resolution of stress during PVR pathogenesis. The study provides further evidence on the utility and applicability of the dispase‐induced mouse model for studying PVR, which can further help in understanding the pathomechanism of the disease, and provide a dynamic system for drug discovery and testing.

## Author contributions

EC, JT and GP designed the experiments, AR, GP and ZS carried out the animal treatment, intravitreal dispase injection and vitreous sample collection, BM and PZ performed the experiments, BM, PZ and EC evaluated the data and prepared the figures and tables, EC wrote the manuscript, and JT and GP reviewed the manuscript. The authors declare they have no conflict of interest.

## Supporting information


**Table S1.** List of proteins identified based upon recorded MS/MS spectra.
